# Shifts in Aboveground Biomass Allocation Patterns of Dominant Shrub Species across a Strong Environmental Gradient

**DOI:** 10.1371/journal.pone.0157136

**Published:** 2016-06-07

**Authors:** Bright B. Kumordzi, Michael J. Gundale, Marie-Charlotte Nilsson, David A. Wardle

**Affiliations:** 1 Department of Forest Ecology and Management, Swedish University of Agricultural Sciences, SE 901 83, Umeå, Sweden; 2 Université Laval, Département des Sciences du bois et de la forêt, Pavillon Abitibi-Price, 2405 rue de la Terrasse, Québec, G1V 0A6, Canada; Chinese Academy of Forestry, CHINA

## Abstract

Most plant biomass allocation studies have focused on allocation to shoots versus roots, and little is known about drivers of allocation for aboveground plant organs. We explored the drivers of within-and between-species variation of aboveground biomass allocation across a strong environmental resource gradient, i.e., a long-term chronosequence of 30 forested islands in northern Sweden across which soil fertility and plant productivity declines while light availability increases. For each of the three coexisting dominant understory dwarf shrub species on each island, we estimated the fraction of the total aboveground biomass produced year of sampling that was allocated to sexual reproduction (i.e., fruits), leaves and stems for each of two growing seasons, to determine how biomass allocation responded to the chronosequence at both the within-species and whole community levels. Against expectations, within-species allocation to fruits was least on less fertile islands, and allocation to leaves at the whole community level was greatest on intermediate islands. Consistent with expectations, different coexisting species showed contrasting allocation patterns, with the species that was best adapted for more fertile conditions allocating the most to vegetative organs, and with its allocation pattern showing the strongest response to the gradient. Our study suggests that co-existing dominant plant species can display highly contrasting biomass allocations to different aboveground organs within and across species in response to limiting environmental resources within the same plant community. Such knowledge is important for understanding how community assembly, trait spectra, and ecological processes driven by the plant community vary across environmental gradients and among contrasting ecosystems.

## Introduction

Plants allocate resources to different organs and this allocation can vary greatly among both plant species and environmental conditions [1, 2]. Currently, the majority of work on allocation patterns among organs has focused on allocation to shoots versus roots (e.g., [3, 4, 5]). However, for aboveground, plants allocate resources to organs that provide different functions, including leaves, stems and reproductive structures [6, 7, 8, 9]. As such, plants vary in their investment in leaf versus stem mass because there is a trade-off between the allocation to leaves for maximizing light capture and thus photosynthetic gain and allocation to stems for hydraulic conductance, supporting plant weight and resisting disturbances [9, 10]. Further, allocation patterns between vegetative and reproductive organs may vary because of trade-offs between the need for carbon gain and the need to produce propagules to maintain future populations [7, 11]. Therefore, analysing different fractions of aboveground biomass (i.e. the proportion of total biomass produced allocated to stem (SMF), leaves (LMF) or reproductive organs (RA)) offers an objective way of linking plant biomass investment to different functions [1, 8] under contrasting environmental conditions.

Over the past decades, most studies of biomass allocation patterns in plants have focused on between-species differences [8, 9, 11]. However, there is growing evidence suggesting that within-species variability of plant traits can represent a significant proportion of total community-level trait variability[12,13,14] and may therefore play an important role in community assembly processes[15,16] and ecosystem functioning [17, 18]. Further, some recent studies have suggested that within-species trait variability constitutes a significant component of plant community responses to local changes in environmental conditions and across environmental gradients [19, 20, 21, 22]. Despite this growing recognition of the role of within-species trait variation as an ecological driver, little is known about within species variability of relative aboveground allocation to different plant organs, in terms of either what drives it or its ecological consequences at the community level. Consequently, assessing variations in biomass allocation patterns within- and between-species can help understand environmental influences on plant community and ecosystem processes.

In this study, we investigated the allocation pattern of aboveground biomass produced during the growing season for the dominant understory plant species on a group of 30 well studied forested lake islands in northern Sweden [23, 24]. These islands differ greatly in size, with larger islands being struck by lightning more often and therefore burning more frequently[23, 25]. Collectively, these islands represent a 5350-year post-fire chronosequence [24]. With increasing time since fire and as islands become smaller, ecosystem retrogression occurs which is characterized by declining soil fertility, standing plant biomass and net primary productivity (NPP), but an increase in light transmission through the forest canopy [24]. As retrogression proceeds along this chronosequence there is also a shift in species dominance in the understory vegetation from *Vaccinium myrtillus* to *Vaccinium vitis-idaea*, and ultimately to *Empertum hermaphroditum*, though with all three species occurring on all islands. This study focuses on these three species and their relative responses to the gradient in terms of biomass allocation. For each of the 30 islands, we measured various biomass allocation variables of the three above mentioned species in order to test three main hypotheses as follows:

We first hypothesize that for each individual species and at the whole community level, relative biomass allocation to sexual reproductive structures (i.e., fruit) will be greatest on small islands, while relative allocation to leaves and stems will be greatest on large islands. This is because small islands have higher light availability and are less fertile, meaning that plants will need to allocate less carbon to vegetative structure needed for light competition (including both leaves and stems to support those leaves), thereby enabling greater allocation to sexual reproductive structures. Second, when species are compared, we hypothesize that relative biomass allocation to sexual reproductive structures (i.e., fruit) will be least for the species best adapted to fertile environments (i.e., *V*. *myrtillus* on large islands) and greatest for those adapted to infertile environments (i.e., *E*. *hermaphroditum* on small islands). This is because we expect that plant species that are best adapted to (and dominate in) fertile environments will need to allocate a greater proportion of biomass production to vegetative organs in order to compete for light, at the expense of sexual reproduction [26]. Finally, we hypothesize that proportional allocation to fruits, leaves and stems will be more responsive to island size for species that are adapted for higher fertility (i.e., *V*. *myrtillus*) because they are less resource conservative and therefore have a higher degree of plasticity. This is consistent with suggestions that plants that are adapted for more favorable conditions are more responsive to variation in soil fertility [27]. In testing these three hypotheses in combination, this study seeks to unravel the adaptive significance of allocation pattern at the within- and between-species, and community level as well as provides insights on how these allocation patterns may be related to relative dominance of species in contrasting environments.

## Materials and Methods

### Ethics Statement

We used 30 forested islands located in the two neighbouring lakes, Hornavan and Uddjaur, in the northern boreal zone of Sweden (65° 55′ N; 17° 43′ E to 66° 09′ N; 17° 55′ E). These islands are not within national reserves and not government protected. The study area is in public lands for which permission is not required from any authority for performing field studies of this type, and our studies did not involve protected or endangered species. We confirm that all national and international rules were complied with during the field work. The research did not involve measurements on humans or animals. The plant material collected for this study was only sampled at a very limited scale and therefore had negligible effects on broader ecosystem functioning. We have no commercial interests or conflicts of interest in performing this work.

### Study system

This study system is a post-fire chronosequence, formed after the retreat of land ice about 9000 years ago [23, 25]. The mean annual precipitation over the past 30 years is 750 mm, and the mean temperature is +13°C in July and -14°C in January. The only major extrinsic factor that varies among islands is the history of lightning-ignited wildfire, with larger islands having burned more frequently than smaller islands because of their larger area to intercept lightning [23, 25]. As such, the islands range in time since the most recent fire from 60 years to 5350 years. Despite this difference in historic disturbance regime, there is no current variation among islands in the disturbance regime that they experience [25, 28]. Previous studies on these islands have shown that as they become smaller and time since fire increases, they enter a state of ‘ecosystem retrogression’ [29] in which there is a reduction in soil fertility (notably a reduced availability of plant-available nitrogen (N) and phosphorus (P)), plant biomass, and ecosystem productivity and an increase in soil N to P ratios [23, 25, 30]. Details on aboveground and belowground properties on these islands are given in S1 Table and macroclimatic measurements over 2011–2013 in S1 Fig.

The overstory vegetation is dominated by *Pinus sylvestris*, *Betula pubescens*, and *Picea abies*, which have their greatest relative biomass on large, medium, and small islands, respectively; these three species constitute >99.8% of all tree individuals. The ground layer vascular vegetation (which is the focus of this study) is dominated by the dwarf shrubs *Vaccinium myrtillus*, *V*. *vitis-idaea*, and *Empetrum hermaphroditum*, which dominate the large, medium and small islands respectively (S1 Table), and which collectively comprise about 98% of this vegetation [24].

### Sample collection and biomass measurements

In the present study, we focused on three dominant vascular species that occur in the understory vegetation, i.e., *V*. *myrtillus* (a deciduous dwarf shrub), *V*. *vitis-idaea* and *E*. *hermaphroditum* (both evergreen dwarf shrubs), because these species each occur on all 30 islands and together constitute nearly all of the understory plant biomass. Further, these understory species are collectively responsible for over half of total net primary productivity in the system [25] and play an important role in the functioning of the boreal forest [31].

For all vegetation measurements and sample collection, we used a 10 m radius plot area on each island established directly adjacent to a set of pre-existing experimental plots used for previous studies on the islands (e.g. [24, 28, 32]). All plots were located at similar distances from the shore regardless of island size to prevent edge and microclimatic effects confounding the results [23, 25] and all measurement were performed twice, in the growing season of both 2012 and 2013.

We measured standing aboveground biomass of each of the three dominant understory species (i.e., *V*. *myrtillus*, *V*. *vitis-idaea*, and *E*. *hermaphroditum*) within each plot during each year at the end of the growing season (i.e., over 12–26 August 2012 and 12–22 August 2013), using the point intercept method, as described by [33] and [24]. Briefly, this method involved counting the total frequency that the vegetation of each species was intercepted by a total of 200 downwardly projected points. The total number of intercepts for each species was then converted to standing biomass per unit area through previously derived calibration equations [24].

In each plot on each island we sampled 20 individual vegetative shoots (i.e., comprising all leaf and stem material) of the three species in 12–26 August 2012 and 12–22 August 2013. Samples were all at least 4 m apart and sourced from different clones. For each of the three species, the shoots were cut at ground level and the portion of the shoot material produced in that growing season (i.e., all new growth occurring between May and July) was separated from shoot material produced during preceding growing seasons. Shoot material produced during the growing season was further separated into leaves and stems. Similarly, we collected all ripe fruits (berries) produced in the same growing season from twenty separate shoots of each of *V*. *myrtillus* and *E*. *hermaphroditum* over 12–26 August in 2012 and 12–22 August 2013 and *V*. *vitis-idaea* over September 8–12 2012 and September 8–12 2013; this later collection for *V*. *vitis-idaea* is because this species produces fruits later in the season than do the other two species. We focused on fruits and not flowers for estimating reproductive allocation because the biomass of flowers is very small relative to that of berries and the use of flowers would therefore greatly underestimate allocation to reproductive material. We then determined the oven-dry weight (60°C, 72 hrs) of all collected vegetative shoot and berry material. Together, these plot level measurements provide data for each species on the proportion of total aboveground material that has been produced during the year of sampling, and the proportion of the year of sampling production allocated to leaf, stem and berry material.

For each plant species on each island in each year, we estimated the reproductive allocation (RA) as the ratio of fruit biomass to the total aboveground biomass (i.e., fruits, stems and leaves) produced in the year of sampling. This measure was used as a measure of the relative amount of aboveground production allocated to sexual reproduction per year [7, 34]. We explored biomass allocation to each of the two vegetative organs (leaves and stem) by estimating the leaf and stem mass fraction (LMF and SMF), which is the fraction of the total aboveground biomass (including fruits) produced in the year of sampling that is allocated to leaves and stems, respectively [1]. For each species on each island we also estimated the annual shoot (i.e., leaf + stem) biomass turnover (hereafter referred to as shoot turnover) as the proportion of total live vegetative shoot biomass produced in the year of sampling.

For each island and each year, we estimated the abundance-weighted community average values of each of the biomass response variables to understand community-level changes in the measured biomass allocation response variables across the island size gradient. These calculations were performed with the method described by [35], separately for each response variable (RA, LMF, SMF and shoot turnover), i.e.,

$$Community\;weighted\;biomass\;allocation\;=\;\sum_{i\;=\;1}^{n}\left(P_{i}*\;{RV}_{i}\;\right)$$

where: *P*_i_ is the proportion of total biomass represented by species *i*, and RV_*i*_ is the estimated value for the response variable of species *i*.

### Environmental data

We used several environmental variables as predictors of the allocation response variables across the 30 islands. Data on the percentage of ambient photosynthetic photon flux density passing through the forest canopy (hereafter ‘light transmission’) was obtained from [24], using measurements obtained from Licor quantum sensors (LI-COR, USA). Briefly, for each island, 50 point measurements were made in a grid of 20 m × 20 m under the tree canopy in the vicinity of the plot used in the present study and about 20 cm above the dwarf shrub layer vegetation. Fifty measurements were simultaneously made in the open (away from the island) and paired with the under canopy measurements to give percentage transmission values. Measurements were performed only on three overcast days. These measures of percentage light transmission were used as a measure of aboveground resource availability for the understory vegetation of each island [36]. Data on soil nutrients (total N and P, and soil mineral N (extractable NH_4_^+^ + NO_3_^-^) and P (PO_4_^-^)) for each island were obtained from [28] and [32]; for each island these measurements were based on composite soil samples collected to 5-cm humus depth throughout the 10 m radius plots on that island. We used data on soil mineral N and P, and the total N to P ratio, as measures of belowground resource availability [37]. For each of the two years of measurement on each island, we used the total number of intercepts of all understory species derived from our point intercept measures (see above) as a measure of total vegetation density which is interpreted as a relative measure of the level of competition exerted by the whole understory plant community [14, 38]. For each year we also used net primary productivity (NPP) of the total understory vegetation on each plot as a predictor, and determined this from our data by using the approach described by [24]. This involved, for each of the three shrub species, multiplying its total aboveground biomass (obtained by converting our point intercept data to biomass using allometric equations; [24]) by the proportion of that biomass which was produced in the year of measurement (using the data from the 20 individual vegetative shoots collected for each species). For each of the two years of measurement on each plot, this value was summed for the three species to provide a measure of NPP for each island.

### Statistical analyses

First, we analysed each of the allocation response variables (RA, LMF, SMF) and shoot turnover) by using a repeated measures split plot ANOVA testing for the effect of island size class (as the main plot factor), species (as the subplot factor), year and their interactive effects, and with islands as the units of replication. When interactions between species or island size and year were significant, we then analysed the data separately for each year using split plot ANOVA testing for island size as the main plot factor and species as the subplot factor. When a significant island size × species interaction was present, one-way ANOVAs were used to assess the effect of island size separately for each species. All significant differences among size classes were further explored by *post hoc* comparisons using Tukey’s test at *P* = 0.05.

For each of the community-weighted response variables, we performed repeated measures ANOVAs testing for the effect of island size class, year and their interactive effect, using islands as the unit of replication. We then analysed this data separately for each year using one-way ANOVA to test the effect of island size class, which were followed by *post hoc* Tukey’s tests at *P* = 0.05 whenever size class effects were detected.

For each year across all islands, we used the Pearson’s correlation coefficients to examine the relationships of response variables (including both allocation variables and shoot turnover) with measured environmental variables for the same islands; this was done both for each species singly and for the community-level measures. The environmental variables included percent light transmission through the forest canopy, total understory shrub density and NPP, soil mineral N and P, and the soil N to P ratio. Multiple regression analysis was used to determine if multiple combinations of environmental variables could best predict different species and community-level biomass allocation response variables, with the most parsimonious model being selected through Akaike information criteria (AIC).

All responses variables were square root transformed to meet the assumption of normality and homogeneity of variance. All statistical analyses were performed using R statistical software [39].

## Results

### Species and community level biomass allocation

**Fig 1:**
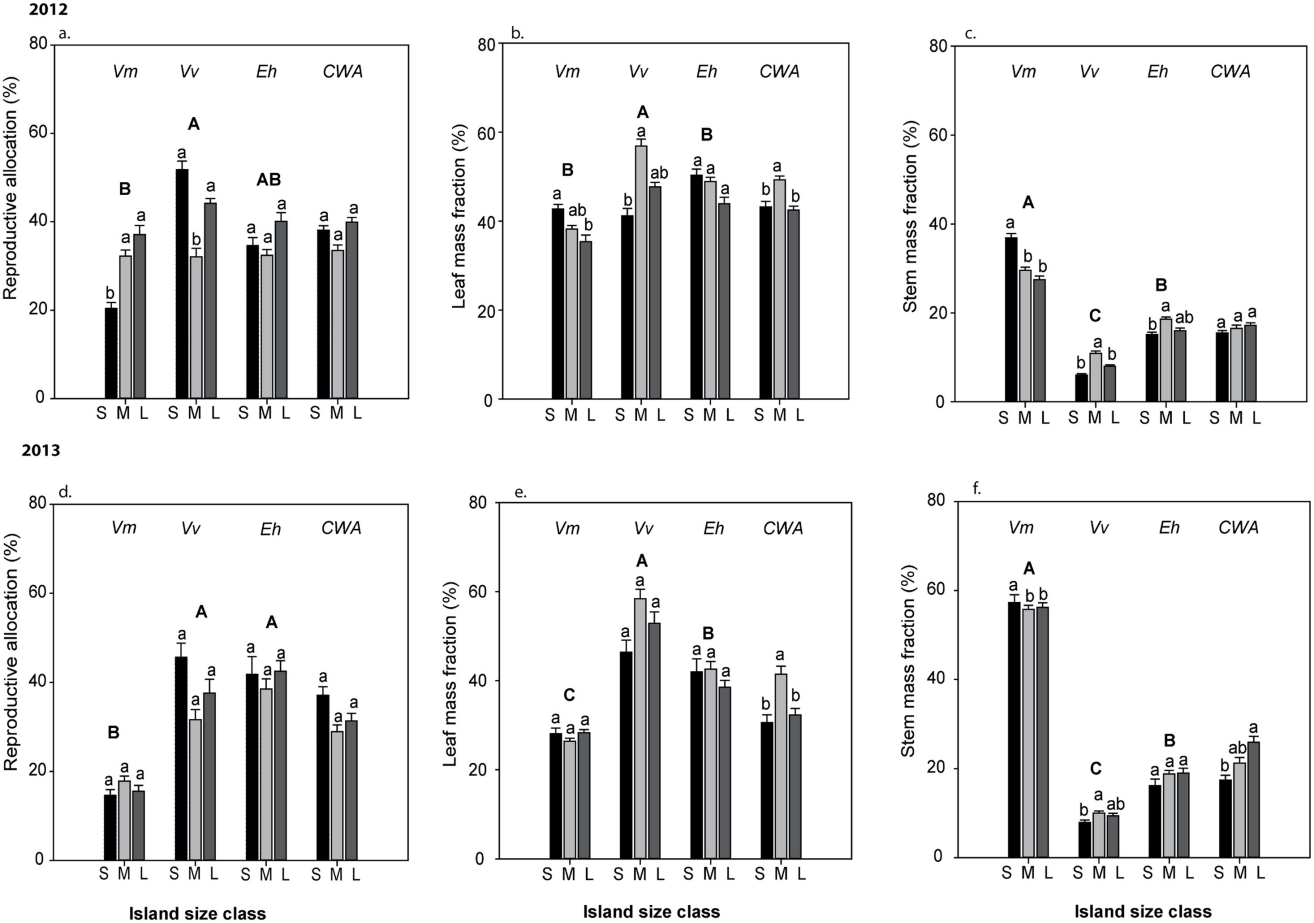
Biomass allocations of the 3 shrubs species, *V*. *myrtillus* (Vm), *V*. *vitis idaea* (Vv) and *E*. *hermaphroditum* (Eh) to different plant organs and their community weighted mean values (CWA) across the island size S, M and L are small, medium and large islands respectively; N = 10 islands of each. Within each group of three bars in each panel, bars topped by the same lower case letter are not significantly different from each other, and groups of three bars topped by the same capital letter are not significantly different among groups (*P = 0*.*05*; Tukey’s test).

**Fig 2:**
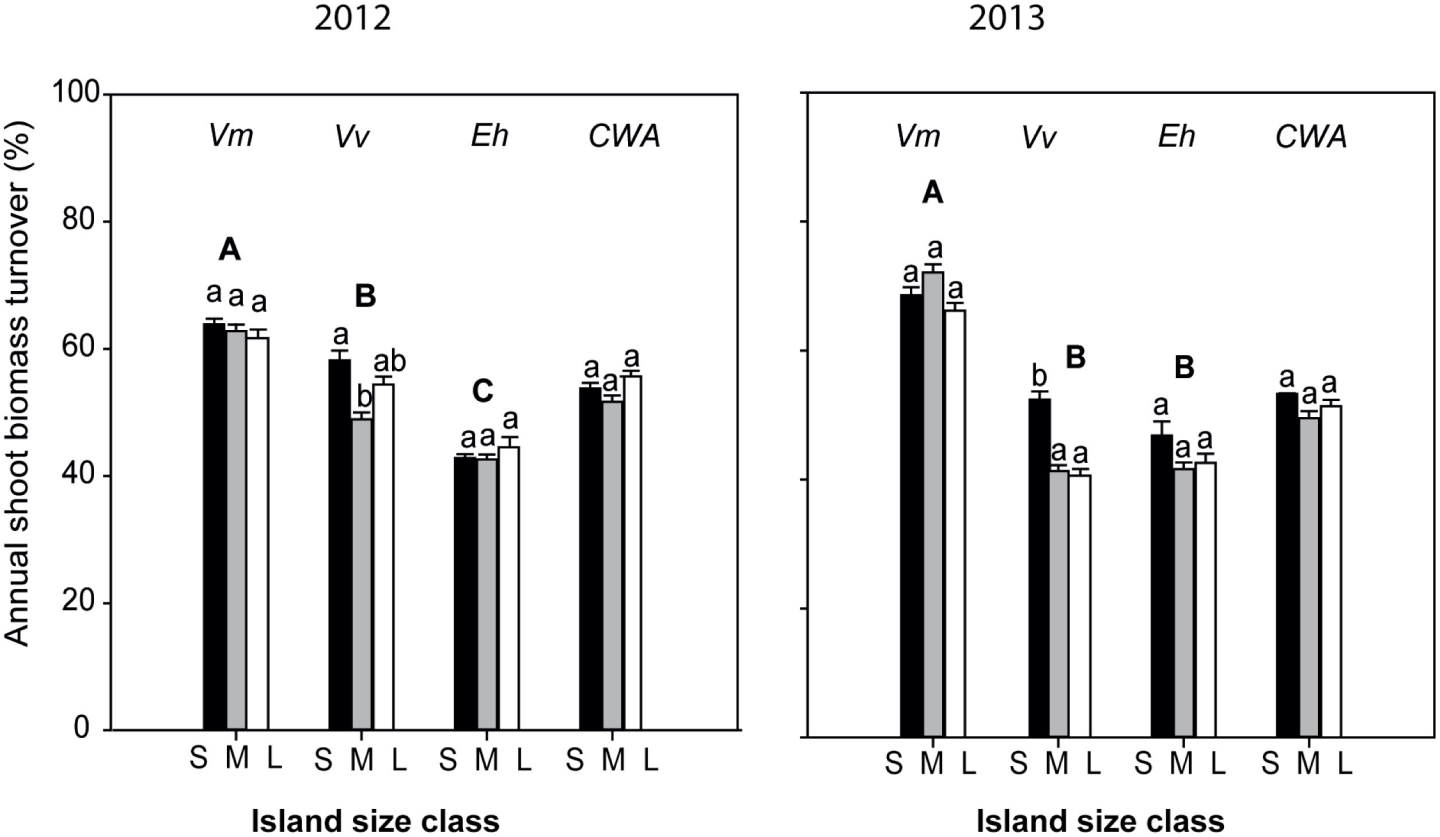
Annual shoot biomass turnover of the 3 shrubs species, *V*. *myrtillus* (Vm), *V*. *vitis idaea* (Vv) and *E*. *hermaphroditum* (Eh) in (a) 2012 and (b) 2013, and their community weighted mean values (CWA) across the island size classes S, M and L are small, medium and large islands respectively; N = 10 islands of each. Within each group of three bars in each panel, bars topped by the same lower case letter are not significantly different from each other, and groups of three bars topped by the same capital letter are not significantly differences among groups (*P = 0*.*05*; Tukey’s test).

The repeated split plot ANOVA showed that all three allocation variables (RA, SMF, LMF), and shoot turnover varied among species and year but not island size class, and were all affected by interactive effect of species by year and species by island size class (Table 1). For data of 2012, ANOVA showed significant effects of species, island size class and their interactive effects on the allocation to reproduction (RA), leaves (LMF) and stems (SMF), and species and its interaction with island size on shoot turnover (S2 Table). Across species in 2012, *V*. *myrtillus* had the lowest values of RA and LMF, and the greatest values of SMF and shoot turnover (Figs 1a, 1b, 1c and 2). Overall RA was greatest on the large islands (mean ± SE = 0.36 ± 0.03, 0.32 ± 0.02 and 0.41 ± 0.02 for small, medium and large islands respectively) while LMF was greatest on the intermediate islands (0.45 ± 0.02, 0.48 ± 0.02 and 0.42 ± 0.02 for small, medium and large islands) as was SMF (0.19 ± 0.02, 0.20 ± 0.02 and 0.17 ± 0.02 for small, medium and large islands). In 2012, interactive effects between species and island size class emerged because RA was least for *V*. *myrtillus* on small islands and for *V*. *vitis-idaea* on intermediate islands, LMF was least for *V*. *myrtillus* on big islands and for *V*. *vitis idaea* on small islands, and SMF was highest for *V*. *myrtillus* on small islands and for the other two species on intermediate islands (Fig 1a–1c).

**Table 1:** Summary of a three-way Analysis of Variance (*F* and *P* values) testing the effect of species, year, island size class and their interactive effects on different biomass allocation response variables; RA, LMF and SMF are the proportion of total shoot biomass produced in the growing season allocated to fruits, leaves and stems, respectively, and shoot turnover is the proportion of total shoot biomass produced in the growing season. Figures in bold indicate statistical significant at *P* < 0.05.

	RA		LMF		SMF		Shoot turnover	
Variables	*F*	*p*	*F*	*p*	*F*	*p*	*F*	*p*
Species	46.07	**<0.000**	**64.88**	**<0.000**	835.26	**<0.000**	46.07	**<0.000**
Year	12.12	**0.001**	18.54	**<0.000**	94.67	**<0.000**	12.12	**0.001**
Island size class (ISC)	1.76	0.191	0.94	0.394	2.61	0.092	1.76	0.191
ISC x Year	1.71	0.184	2.05	0.149	2.26	0.108	1.71	0.184
Species x Year	12.43	**<0.000**	13.04	**<0.000**	68.17	**<0.000**	12.43	**<0.000**
ISC x Species	5.92	**0.000**	5.21	**0.001**	5.81	**<0.000**	5.92	**<0.000**
ISC x species x Year	0.71	0.584	0.41	0.803	1.30	0.273	0.71	0.584

Degree of freedom for Island size class = 2, 27; Year = 1, 135, Species = 2, 135; Island size class x Species = 4, 135; Year x Island size class x Species = 4, 135

For shoot turnover, interactive effects between species and island size class occurred because only *V*. *vitis idaea* was responsive to island size though being least on the intermediate islands (Fig 2). For 2013, ANOVAs showed a strong species effect for all response variables, but an effect of island size and its interaction with species only for shoot turnover (S2 Table, Figs 1d–1f and 2b). Variation among species in 2013 reflected those in 2012. Further, in 2013 shoot turnover was overall greatest on small islands and was most responsive to island size for *V*. *vitis-idaea* (Fig 2).

At the community level, the repeated measures ANOVA showed that community weighted averages (CWAs) of the all three allocation variables (RA, SMF, LMF), and shoot turnover differed among years, with LMF affected by island size class and SMF affected by the interactive effect of year with size class (Table 2). For 2012, ANOVA showed a significant effect of island size on LMF (Fig 1b, S3 Table), whereas for 2013, island size had significant effects on both LMF and SMF (Fig 1e and 1f; S3 Table). Overall, LMF was greatest on intermediate islands for both years and SMF was greatest on large islands in 2013 (Fig 1).

**Table 2:** Summary of a two-way Analysis of Variance (*F* and *P* values) testing the effect of island size class, year, and their interactive effects on different community-weighted biomass allocation response variables. RA, LMF and SMF are the proportion of total shoot biomass produced in the growing season allocated to fruits, leaves and stems, respectively, and shoot turnover is the proportion of total shoot biomass produced in the growing season. Figures in bold indicate statistical significant at *P* < 0.05.

	RA		LMF		SMF		Shoot turnover	
Variables	*F*	*p*			*F*	*p*	*F*	*p*
Island size class (ISC)	2.18	0.132	5.445	**0.010**	2.47	0.104	1.58	0.225
Year	10.27	**0.003**	49.31	**<0.000**	38.58	**<0.000**	4.76	**0.038**
ISC x Year	2.22	0.128	0.89	0.422	5.58	**0.001**	1.10	*0*.*349*

Degree of freedom for Island size class = 2, 27; Year = 1, 27: Island size class x Year = 2, 27

### Relationship with environmental variables

**Fig 3:**
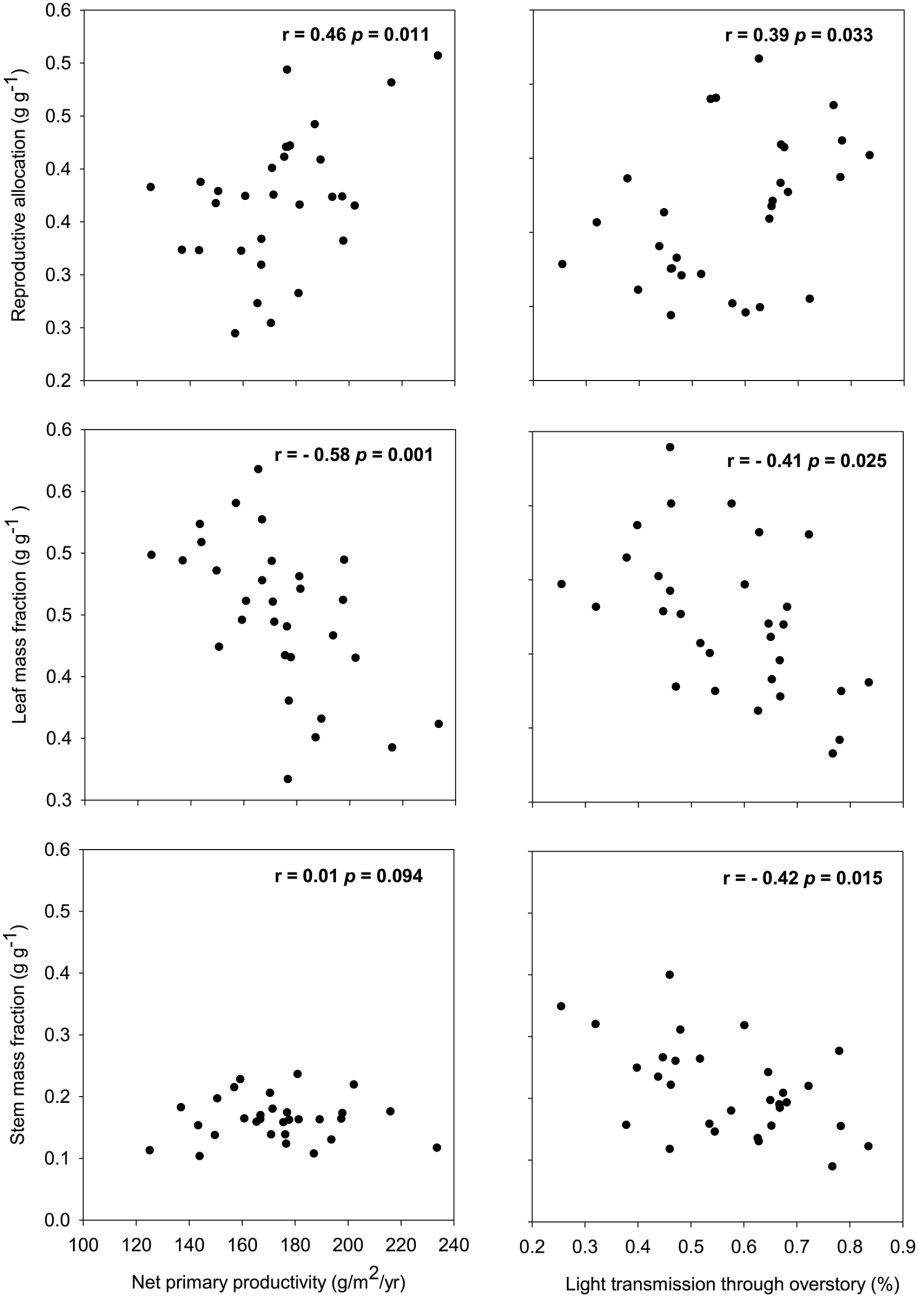
The relationships between community-weighted averages of dwarf shrub biomass allocation response variables (reproductive allocation, leaf mass fraction, and stem mass fraction) and net primary productivity for 2012 and light availability for 2013 across the 30 islands.

When univariate correlation analyses were performed for 2012, several significant relationships occurred between *V*. *myrtillus* response variables and the environmental variables. Values of RA and SMF were significantly positively related to soil mineral P, while RA and SMF were negatively and positively related to soil N:P respectively (S4 Table). Net primary productivity (NPP) was positively correlated with RA, but negatively correlated with LMF, SMF and shoot turnover (S4 Table). For *V*. *vitis idaea*, RA, LMF and shoot turnover were significantly positively correlated with light availability while SMF was negatively correlated with it; shoot turnover was also positively correlated with NPP (S4 Table). However, for *E*. *hermaphroditum* the only significant relationships were for soil mineral P which was negatively correlated with LMF and positively correlated with shoot turnover (S4 Table). The community weighted average (CWA) value of RA and shoot turnover was positively correlated with NPP whereas LMF was negatively correlated with it (S4 Table, Fig 3).

For 2013, the response variables of *V*. *myrtillus* were unrelated to the environmental variables except that NPP was positively correlated with LMF but negatively correlated with SMF (S5 Table). For *V*. *vitis idaea*, light availability was negatively related to SMF and positively related to shoot turnover (S5 Table). For *E*. *hermaphroditum*, soil mineral N was negatively related to RA but positively correlated with SMF (S5 Table,). For the CWA measures, RA was positively related to light availability, while LMF and SMF were negatively correlated with it (Fig 3). Further, the CWA of SMF was negatively correlated with soil N:P (S5 Table).

Our multiple regression analyses of the data revealed that, including more than one predictor variable in the regression was necessary for best model fit in only a minority of instances in 2012 (Table 3). As such, for *V*. *myrtillus* in 2012, model fits were best for RA and LMF when understorey shrub density and NPP were included as predictors, for SMF with soil mineral P and soil N:P as predictors, and for shoot turnover with N:P and NPP as predictors (Table 3). Further, model fit was best for shoot turnover of *V*. *vitis-idaea* when light availability, soil N:P and NPP were included in the model (Table 3). However, for 2013, model fit was never improved by including more than one predictor in the regression model (S5 Table).

**Table 3:** Results from multiple stepwise regression testing relationships between species biomass allocation responses and environmental variables for 2012. For each response variable the most parsimonious model is presented based on Akaike Information Criteria (AIC). Values of *t* are shown for each selected response variable, along with adjusted *R*^2^ and *P* values for the regression models. For each of the response variables, the presented models contain only those variables for which *t*-values are shown.

			Environmental variables (t-values)								
	Response variables	Light	USD	Mineral N	Mineral P	Soil N:P	NPP	Df	AIC	*Adjusted R*^*2*^	*P*
*V*. *myrtillus*	RA	-	2.27*	-	-	-	2.30*	25	-148.62	0.44	**< 0.000**
	LMF	-	-2.16*	-	-	-	-3.39**	27	-170.43	0.32	**0.002**
	SMF	-	-	-	-2.11*	2.61*	-	26	-179.03	0.36	**0.002**
	Turnover	-	-	-	-	2.08*	4.23***	26	-181.52	0.39	**0.001**
*V*. *vitis-idaea*	RA	2.59*	-	-	-	-	-	28	-142.99	0.16	**0.015**
	LMF	2.14*	-	-	-	-	-	28	-158.76	0.11	**0.042**
	SMF	- 4.26***	-	-	-	-	-	28	-227.78	0.37	**< 0.000**
	Turnover	2.53*	-	-	-	2.81**	4.02***	25	-170.19	0.53	**< 0.000**
*E*. *hermaphroditum*	LMF	-	-	-	-2.48*	-	-	28	-159.04	0.15	**0.019**
	Turnover	2.33 *	-	-	-	-	-	26	-183.53	0.27	**0.011**
Community weighted average	RA	-	-	-	-	-	2.54*	27	-168.53	0.22	**0.012**
	LMF	-	-	-	-	-	- 4.36***	27	-177.75	0.37	**0.001**
	Turnover	-	-	-	-	-	4.86***	27	-193.01	0.43	**0.000**

Significance level for selected variables:

*** *P*<0.001,

** *P*<0.01,

* *P*<0.05,

^#^ *P*<0.10.

Significant *P*-values (<0.05) associated with the regressions are in bold. Response variables for which no variables were significant are not included in this table.RA, LMF and SMF = proportion of total shoot biomass produced in the growing season allocated to fruits, leaves and stems respectively. Turnover = Proportion of total shoot biomass produced in the growing season. Light = Light transmission through the overstory canopy across the 30 islands (%). Mineral N = Mineral N (mg.g^-1^). Mineral P = Mineral P (mg.g^-1^). Soil N:P = ratio of soil N to P. USD = Understorey shrub density (intercepts per 200 points). NPP = Net primary productivity of shrubs (g.m^-2^ yr^-1^).

## Discussion

Our study shows directional shifts in shoot biomass allocation among organs of dominant dwarf shrub species across a strong environmental gradient, both at the within-species and whole community levels. Further, coexisting shrub species with contrasting ecological strategies differed greatly both in terms of allocation pattern and how this pattern responded to the same environmental gradient. We now discuss our findings and their implications at the species, community and ecosystem levels.

Our first hypothesis that biomass allocation to reproductive structures (i.e., fruits) would be greatest on small islands at both the within-species and whole community-levels was not supported. For instance, for 2012, RA was least on small islands for *V*. *myrtillus* and on medium islands for *V*. *vitis-idaea*, and did not change with island size class for *E*. *hermaphroditum*. Our results from the correlation and multiple regression analysis suggest that at least in 2012, RA for *V*. *myrtillus* was most closely and positively linked to soil nutrient status, NPP and understory density. Soil fertility increases with island size, meaning that NPP and vegetation density (and hence plant competition) is high on the large islands on which *V*. *myrtillus* dominates [14, 28]. The higher RA of this species on large islands suggests that greater investment in sexual reproductive structures may contribute to it maintaining its dominance in the most competitive environment [14, 24]. In contrast, the RA of *V*. *vitis-idaea* was most closely related to light availability, which was least on the medium islands in which this species dominates. Instead, this species allocates most of its biomass to leaves and stems in the lowest light environment, presumably in order to maximize light capture. These contrasting results suggest that shoot allocation patterns respond in contrasting ways for different species to the same environmental factors, in line with optimal biomass allocation theory that each species should prioritize resource allocation to organs that maximises its own survival and fitness [3, 8].

At the community level, we found that for both growing seasons LMF was greatest on medium islands. This result was reflective of the response of *V*. *vitis-idaea*, which is most abundant on medium islands [14, 23, 24] and which allocated a greater proportion of its biomass to leaves on these islands. This finding highlights the important contribution of within-species variability to observed community-level responses across environmental gradients [14, 19]. Further, the strong negative relationships observed of LMF with both NPP and light availability at the community level (Fig 3) suggest that changes in availability of resources may exert a direct influence on community-level patterns of allocation [14, 30]. Taken together, our results suggest that both species turnover and within-species variability in biomass allocation contribute significantly to community-level responses to variation of extrinsic environmental factors [13], [19].

Across species, we found RA was least in *V*. *myrtillus* (dominant on fertile large islands) and with no difference between *V*. *vitis-idaea* and *E*. *hermaphroditum* (dominant on medium and the least fertile small islands respectively), providing only partial support for our second hypothesis (i.e. that biomass allocation to sexual reproductive structures would be least for species that are more adapted for fertile environments). *Vaccinium myrtillus* consistently allocated the greatest biomass to stems, whereas *V*. *vitis-idaea* allocated more biomass to leaves, indicating contrasting strategies for how two dominant and competing coexisting species invest in order to access light in relatively fertile low-light environments. As such, the strategy for *V*. *vitis-idaea* (which is evergreen) is to invest heavily in long-lived leaves, whereas *V*. *myrtillus* (which is deciduous) annually produces highly efficient photosynthetic leaves [30] while investing more in taller, longer lived stems to support the leaves. This finding that *V*. *myrtillus* allocated greater biomass to stems than did the other species may enable it to reduce self-shading, which could help it compete better for light against other species in low light environments [7, 9]. Our results support recent suggestions that co-existing plant species can display highly contrasting biomass distributions to shoot organs to achieve optimum resource acquisition within a single plant community [40].

Finally, we found support for our third hypothesis that proportional biomass allocation to reproduction, leaves and stems would be more responsive to the island size gradient (and thus variation in soil fertility) for species that dominate on larger islands. As such, biomass allocation patterns of *V*. *myrtillus* and *V*. *vitis-idaea* were more responsive to island size than were those of *E*. *hermaphroditum*. For the two *Vaccinium* species, the plasticity of traits such as RA, LMF and SMF could contribute to maintaining or increasing fitness of these species under contrasting environments including those for which they are poorly adapted [41], and may constitute an integral part of the mechanism of resource acquisition in productive environments [42]. Further, the fact that *V*. *myrtillus* and *V*. *vitis-idaea* are abundant in relatively productive environments could be because high trait plasticity enables these species to optimize their allocation in response to high levels of competition from coexisting species [2, 43, 44]. In total, our findings support the idea that within-plant allocation for resource-acquisitive species is more responsive to variation in resource availability than is the case for resource-conservative ones because they are superior in exploiting resources across a wider range of resource availability including in conditions for which they are least well adapted [2, 42].

## Conclusion

Our study provides important insights into the mechanisms underlying plant biomass adaptive allocation patterns, and this has implications for better understanding of community assembly and ecosystem productivity. First, contrary to some widely accepted assumptions that between-species trait variability is the major contributor to whole-community responses to environmental factors, we show that within-species variability in biomass allocation responses is an essential component of community response, and is consistent with recent calls to incorporate within-species trait variability into studies of community assembly [12, 13, 20]. Second, we show greater within-species responsiveness of allocation to environmental variables for species adapted to more productive and competitive environments, thus providing evidence for the hypothesis that the within-species plant trait responses to extrinsic factors is related to plant resource acquisition strategy [27, 43]. Third, our study shows that coexisting species in a single community can vary greatly in their biomass allocation patterns to aboveground organs as a means to achieve optimum resource acquisition [1, 40]. These contrasting strategies especially between dominant species can make an important contribution to the whole community level response to changes in resource availability. Finally, our study suggests that knowledge about how within- and between-species variability contribute to community level patterns could lead to an improved understanding on how community assembly, trait spectra, and ultimately ecosystem processes driven by the plant community, vary across environmental gradients and among contrasting ecosystems.

## Supporting Information

S1 Fig Mean monthly precipitation and temperature recorded at the study area over 2011–2013. (TIF)

S1 Table Measurements of selected ecosystem properties (mean values ± standard errors) across the island size gradient. (DOCX)

S2 Table Summary of separate two-way Analysis of Variance (*F* and *P* values) analyses testing the effect of species, island size class and their interactive effects on different biomass allocation response variables for each of 2012 and 2013. (DOCX)

S3 Table Summary of separate one-way Analysis of Variance (*F* and *P* values) testing the effect of island size class on different community-weighted biomass allocation response variables for each of 2012 and 2013. (DOCX)

S4 Table Pearson’s correlation coefficients between allocation response variables (at both the species and whole community levels) and environmental variables across the 30 islands for 2012. (DOCX)

S5 Table Pearson’s correlation coefficients between species and community-level biomass allocation responses and environmental variables across the 30 islands for the year 2013. (DOCX)
