# Supplementary figures and images for: Shifts in Aboveground Biomass Allocation Patterns of Dominant Shrub Species across a Strong Environmental Gradient
Source: PLoS One. 2016 Jun 7;11(6):e0157136. doi: 10.1371/journal.pone.0157136 (PMC4896472; doi:10.1371/journal.pone.0157136)

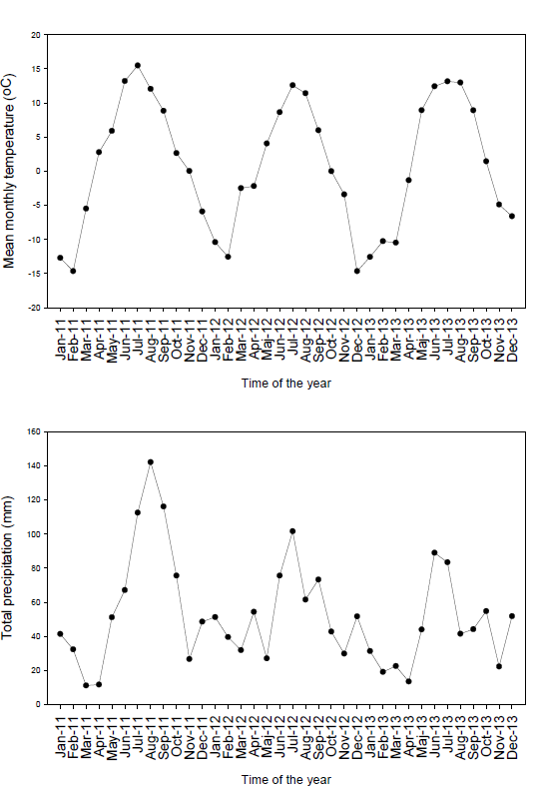

Supplement: S1 Fig — (TIF) [file pone.0157136.s001.tif]
