# Supplementary material for: Shifts in Aboveground Biomass Allocation Patterns of Dominant Shrub Species across a Strong Environmental Gradient
Source: PLoS One. 2016 Jun 7;11(6):e0157136. doi: 10.1371/journal.pone.0157136 (PMC4896472; doi:10.1371/journal.pone.0157136)
Supplement: S1 Table — (DOCX) [file pone.0157136.s002.docx]

**S1 Table**. Measurements of selected ecosystem properties (mean values ± standard errors) across the island size gradient. Within each row, numbers followed by the same letter are not statistically significant at *P* = 0.05 (Tukey’s test following one-way ANOVA). Data from Wardle *et al*. (1997, 2003, 2004, 2012) and Wardle and Zackrisson (2005).

| Ecosystem property |  | Island size |  |
| --- | --- | --- | --- |
|  | Small | Medium | Large |
| Time since last fire (years) | 3250 ± 439 a | 2180 ± 385 b | 585 ± 233 c |
| Net primary productivity (g/m^2^/yr) | 159 ± 18 b | 247 ± 12 a | 256 ± 14 a |
| Standing plant biomass (g/m^2^) | 3470 ± 470 b | 8340 ± 877 a | 9349 ± 485 a |
| Vascular plant species richness^a^ | 10.6 ± 0.6 a | 8.6 ± 0.4 b | 6.6 ± 0.6 c |
| Humus C to N ratio | 32.9 ± 0.79 b | 36.0 ± 1.17 ab | 40.4 ± 1.18 a |
| Humus C to P ratio | 759 ± 30 a | 687 ± 36 ab | 623 ± 20 b |
| Humus N to P ratio | 23.3 ± 1.1 a | 19.1 ± 0.9 b | 15.4 ± 0.5 c |
| Mineral N (MIN) (µgN/g) | 25.3 ± 8.0 b | 58.1 ± 9.2 a | 38.2 ± 14.4 ab |
| Dissolved org N (DON) (µgN/g) | 40.3 ± 4.6 b | 50.7 ± 5.5 a | 39.1 ± 7.2 b |
| MIN/(MIN+DON) | 0.39 ± 0.03 b | 0.53 ± 0.05 a | 0.49 ± 0.04 a |
| Mineral P (µgP/g) | 24.4 ± 2.3 b | 37.7 ± 4.3 a | 43.6 ± 4.9 a |
| Membrane-extractable P (mmol/kg) | 4.9 ± 0.3 b | 6.5 ± 0.4 a | 5.9 ± 0.7 ab |
| Light transmission (%) | 68.6 ± 2.6 a | 47.1 ± 3.7 b | 55.8 ± 4.5 ab |

^a^Number of species in a 10 m radius circular plot
