# Supplementary material for: Shifts in Aboveground Biomass Allocation Patterns of Dominant Shrub Species across a Strong Environmental Gradient
Source: PLoS One. 2016 Jun 7;11(6):e0157136. doi: 10.1371/journal.pone.0157136 (PMC4896472; doi:10.1371/journal.pone.0157136)
Supplement: S2 Table — (DOCX) [file pone.0157136.s003.docx]

**S2 Table**. Summary of separate two-way Analysis of Variance (*F* and *P* values) analyses testing the effect of species, island size class and their interactive effects on different biomass allocation response variables for each of 2012 and 2013. RA, LMF and SMF are the proportion of total shoot biomass produced in the growing season allocated to fruits, leaves and stems, respectively, and shoot turnover is the proportion of total shoot biomass produced in the growing season. Figures in bold indicate statistical significant at *P* < 0.05.

|  |  | **RA** | |  | **LMF** | |  | **SMF** | |  | **Shoot turnover** | |
| --- | --- | --- | --- | --- | --- | --- | --- | --- | --- | --- | --- | --- |
| Year | Variables | *F* | *P* |  | *F* | *P* |  | *F* | *P* |  | *F* | *P* |
| 2012 | Island size class (ISC) | 4.82 | **0. 016** |  | 3.61 | **0.041** |  | 5.01 | **0.014** |  | 1.43 | 0.257 |
|  | Species | 17.29 | **<0.000** |  | 19.02 | **<0.000** |  | 406.95 | **<0.000** |  | 116.17 | **<0.000** |
|  | Species*ISC | 10.01 | **<0.000** |  | 6.13 | **0.001** |  | 11.13 | **<0.000** |  | 2.83 | **0.033** |
|  |  |  |  |  |  |  |  |  |  |  |  |  |
| 2013 | Island size class (ISC) | 0.35 | 0.706 |  | 0.85 | 0.44 |  | 1.55 | 0.301 |  | 3.85 | **0.034** |
|  | species | 30.68 | **<0.000** |  | 44.99 | **<0.000** |  | 432.86 | **<0.000** |  | 158.36 | **<0.000** |
|  | Species*ISC | 1.00 | 0.414 |  | 1.24 | 0.306 |  | 0.61 | 0.656 |  | 4.23 | **0.005** |

Degree of freedom for Island size class = 2, 27; Species = 2, 54: Island size class x Species = 4, 54.
