# Supplementary material for: Shifts in Aboveground Biomass Allocation Patterns of Dominant Shrub Species across a Strong Environmental Gradient
Source: PLoS One. 2016 Jun 7;11(6):e0157136. doi: 10.1371/journal.pone.0157136 (PMC4896472; doi:10.1371/journal.pone.0157136)
Supplement: S5 Table — (DOCX) [file pone.0157136.s006.docx]

**S5 Table.** Pearson’s correlation coefficients between species and community-level biomass allocation responses and environmental variables across the 30 islands for the year 2013. RA, LMF and SMF are the proportion of total shoot biomass produced in the growing season allocated to fruits, leaves and stems respectively, and shoot turnover is the proportion of total shoot biomass produced in the growing season.

|  |  | **RA** | |  | **LMF** | |  | **SMF** | |  | **Shoot turnover** | |
| --- | --- | --- | --- | --- | --- | --- | --- | --- | --- | --- | --- | --- |
| **Species** | **Environmental variables** | *r* | *P* |  | *r* | *P* |  | *r* | *P* |  | *r* | *P* |
| *V. myrtillus* | Light | 0.08 | 0.673 |  | - 0.01 | 0.964 |  | - 0.07 | 0.698 |  | 0.18 | 0.334 |
|  | Understorey Shrub density | 0.11 | 0.546 |  | - 0.14 | 0.477 |  | - 0.02 | 0.934 |  | 0.31 | 0.*091* |
|  | Mineral N | - 0.36 | 0.051 |  | 0.25 | 0.182 |  | 0.17 | 0.356 |  | - 0.35 | 0.*057* |
|  | Mineral P | - 0.08 | 0.660 |  | 0.07 | 0.711 |  | 0.03 | 0.868 |  | - 0.25 | 0.182 |
|  | Soil N:P | - 0.04 | 0.360 |  | - 0.32 | 0.*082* |  | 0.28 | 0.141 |  | 0.31 | 0.*091* |
|  | NPP | 0.11 | 0.568 |  | 0.42 | **0.020** |  | - 0.42 | **0.022** |  | - 0.21 | 0.267 |
|  |  |  |  |  |  |  |  |  |  |  |  |  |
| *V. vitis idaea* | Light | 0.28 | 0.132 |  | - 0.23 | 0.222 |  | - 0.46 | **0.009** |  | 0.53 | **0.002** |
|  | Understorey Shrub density | - 0.34 | 0.*065* |  | 0.34 | 0.069 |  | 0.28 | 0.129 |  | 0.08 | 0.689 |
|  | Mineral N | 0.06 | 0.759 |  | - 0.08 | 0.656 |  | 0.08 | 0.679 |  | - 0.17 | 0.378 |
|  | Mineral P | 0.32 | 0.*089* |  | 0.33 | 0.*073* |  | 0.17 | 0.369 |  | - 0.34 | 0.673 |
|  | Soil N:P | 0.19 | 0.327 |  | - 0.18 | 0.329 |  | - 0.15 | 0.434 |  | 0.40 | **0.029** |
|  | NPP | - 0.27 | 0.147 |  | 0.29 | 0.118 |  | 0.12 | 0.544 |  | 0.08 | 0.665 |
|  |  |  |  |  |  |  |  |  |  |  |  |  |
| *E. hermaphroditum* | Light | 0.16 | 0.401 |  | - 0.04 | 0.820 |  | 0.33 | 0.*075* |  | 0.26 | 0.169 |
|  | Understorey Shrub density | 0.08 | 0.669 |  | - 0.08 | 0.669 |  | - 0.06 | 0.766 |  | 0.06 | 0.760 |
|  | Mineral N | - 0.42 | **0.019** |  | 0.30 | 0.113 |  | 0.54 | **0.002** |  | - 0.28 | 0.139 |
|  | Mineral P | 0.11 | 0.564 |  | 0.22 | 0.247 |  | 0.13 | 0.497 |  | 0.10 | 0.588 |
|  | Soil N:P | - 0.06 | 0.707 |  | 0.17 | 0.377 |  | 0.17 | 0.362 |  | 0.08 | 0.667 |
|  | NPP | 0.16 | 0.402 |  | - 0.22 | 0.236 |  | - 0.01 | 0.951 |  | 0.14 | 0.457 |
|  |  |  |  |  |  |  |  |  |  |  |  |  |
| Community weighted average | Light | 0.41 | **0.025** |  | - 0.41 | **0.025** |  | - 0.42 | **0.015** |  | 0.29 | 0.115 |
|  | Understorey Shrub density | - 0.19 | 0.315 |  | 0.30 | 0.111 |  | - 0.02 | 0.911 |  | 0.13 | 0.485 |
|  | Mineral N | - 0.22 | 0.249 |  | 0.22 | 0.253 |  | 0.30 | 0.104 |  | - 0.22 | 0.252 |
|  | Mineral P | - 0.19 | 0.311 |  | 0.26 | 0.166 |  | 0.11 | 0.564 |  | 0.16 | 0.395 |
|  | Soil N:P | 0.23 | 0.221 |  | - 0.14 | 0.451 |  | - 0.39 | **0.035** |  | - 0.04 | 0.836 |
|  | NPP | - 0.18 | 0.338 |  | 0.17 | 0.361 |  | 0.07 | 0.732 |  | 0.30 | 0.112 |

Light = Light transmission through the overstory canopy (%). Understory shrub density = Total number of intercepts per 200 points. NPP = Net primary productivity of shrubs (g.m^-2^ yr^-1^).
